# Diastereoselective Synthesis of Cyclopentanediols by InCl_3_/Al Mediated Intramolecular Pinacol Coupling Reaction in Aqueous Media

**DOI:** 10.3390/molecules13102652

**Published:** 2008-10-27

**Authors:** Yunhua Chen, Jieping Wan, Chunyan Wang, Cuirong Sun

**Affiliations:** 1 Department of Chemistry, Zhejiang University, Hangzhou, 310027, P.R. China; 2 Zhejiang Hisun Pharm. Co. Ltd. Taizhou, 318000, P.R. China

**Keywords:** Intramolecular, Pinacol coupling, Indium, Aluminium, Aqueous media

## Abstract

A “green” and practical intramolecular pinacol coupling reaction promoted by InCl_3_/Al catalysts in aqueous media has been developed. Under mild conditions, a novel class of polysubstituted cyclopentane-1,2-diols have been obtained with excellent diastereoselectivity.

## Introduction

In organic synthesis, the formation of carbon-carbon bonds is one of the most fundamental and important operations in the construction of functional molecules. Pinacol coupling, which involves the homo- or cross reductive dimerization of two carbonyls represents one of the most classical ways of generating carbon-carbon bonds [1]. Since its discovery in 1859 [2], the pinacol coupling reaction has been a focal point in synthetic chemistry not only for its carbon-carbon bond generating power, but also for the wide utility of the 1,2-diols obtained from these reactions. These 1,2-diols are valuable synthons in the synthesis of biologically active natural products or molecular fragments [3,4].

After the first report over a century ago, numerous modified pinacol coupling methods have been developed. Instead of the early use of sodium as catalyst, a variety of other metals or metal/Lewis acids such as Zn [5], Mg [6], Ti [7], Sm [8,9], Cr [10], In [11], and VCl_3_/Al [12], etc. have been proven to be efficient metal sources for pinacol coupling reactions. Hirao *et al*. [12] reported the main pinacol coupling protocol that is successful in water. Recently, another water-mediated pinacol coupling process has been reported by Hosono and co-workers [13]. However, most of the reported catalyst systems have unfavorable properties due to the toxicity of the metal catalyst(s) and/or the use of toxic organic solvents as reaction media. Therefore, environmentally benign methodologies to produce this valuable reaction are highly desirable. Unlike the corresponding intermolecular reactions, intramolecular pinacol coupling reactions furnish cyclized diols, which usually limits their applications [14]. Much fewer systematic intramolecular pinacol coupling methods are available than their intermolecular counterparts [15,16].

Indium as a nontoxic metal has been extensively studied due to its versatile catalytic activity and tolerance to water and air. A number of organic reactions have been successfully achieved in water or in aqueous media employing indium or indium salts as catalysts [17,18]. It is also our interest to explore broader application of indium as a practical metal catalyst [19,20,21]. In our previous work studying indium catalysis, we developed InCl_3_/Al catalyzed pinacol coupling reactions in aqueous media, in which a class of vicinal diols derived from aromatic aldehydes, ketones and aldehyde-ketone cross coupling have been synthesized in good yields [22]. In order to further explore the scope of this catalyst system, we have also investigated the intramolecular coupling reactions of 1,5-dicarbonyl substrates, and we present here our results from this study.

## Results and Discussion

### Screening of reaction temperature

Based on the results of our intermolecular pinacol coupling method, we applied the InCl_3_/Al co-catalyst system to the intramolecular coupling reaction of 1,3,5-triphenylpentane-1,5-dione, which contains a 1,5-dicarbonyl skeleton. At the presence of 1 equivalent mol InCl_3 _(0.5 mol scale) and 3.5 equivalent mol of aluminum powder, the substrate was heated at 80 ^o^C for 8 h in 4 mL EtOH/H_2_O (1:1) to gave the target diol **2a** in 51 % isolated yield. We then optimized the temperature on this model reaction, and found that at 70 ^o^C, the pinacol coupling product was formed as the main product after 8 h, and it could be isolated in 65 % yield with column chromatography on silica gel. It is noteworthy that at room temperature, the reaction also proceeded well, but much longer time is required for satisfactory conversion. 

### Investigation of the scope of the InCl_3_/Al catalyzed intramolecular pinacol coupling

With the proper conditions in hand, we than turned to examine the scope of application of this protocol for the intramolecular pinacol coupling reaction. A group of 1,5-dicarbonyl substrates analogous to 1,3,5-triphenylpentane-1,5-diketone were subjected to the reaction. The representative results are summarized in Table 1. Symmetrical aromatic 1,5-diketones with diversified functional groups furnished the corresponding five-membered vicinal 1,2-diols in moderate to good yields. While alkyl, alkoxy as well as halogen substitutions were tolerated by this catalyst system, the amino group didn’t give the expected product. Unsymmetrical 1,5-diketones and 1,4-ketones (entries 13 and 14) were also subjected to the reaction conditions, but unfortunately none of the corresponding pinacols were observed under the standard conditions. What should be noted is that most of the substrates gave the corresponding diols with excellent diastereoselectivity, as only one diastereoisomer was observed after chromatography purification. However, the highly symmetrical structure of the products and the lack of vicinal hydrogen atom make it impossible to identify their relative configuration by ^1^H-NMR [23]. Further work on this issue is presently in progress in our group. 

**Figure 1:**
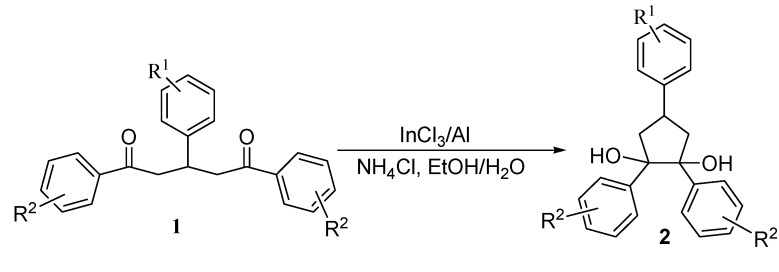
Intramolecular pinacol coupling of aromatic 1,5-diketones.

**Table 1:** InCl_3_/Al mediated intramolecular pinacol coupling or various aromatic 1,5-diketones.^a^

Entry	Diketone		Product	Yield (%)^b^	d. e. (%)^c^
	R_1_	R_2_			
1	H	H	**2a**	65	> 99
2	H	4-CH_3_	**2b**	78	> 99
3	H	4-CH_3_O	**2c**	75	58
4	H	4-Cl	**2d**	62	> 99
5	4-CH_3_	H	**2e**	80	> 99
6	4-Cl	H	**2f**	83	> 99
7	4-CH_3_O	H	**2g**	88	> 99
8	2-Cl	H	**2h**	61	44
9	4-CH_3_	4-CH_3_	**2i**	90	> 99
10	4-F	H	**2j**	79	> 99
11	4-N(CH_3_)_2_	H	**-**	trace	-
12	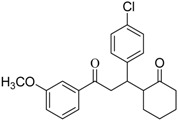		**-**	-	-
13^d^					
14^e^	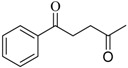		nr		

^a^ Reaction conditions: 0.5 mmol **1**, 0.5 mmol InCl_3_, 1.8 mmol aluminum powder and 0.5 g NH_4_Cl mixed in 4 mL solvent (V_EtOH_:V_H2O_=1:1) and stirred at 70 ^o^C for 8 h. ^b ^Isolated yield. ^c ^Diastereomeric excess was calculated from the ^1^H-NMR of products based on clearly distinguishable signals. ^d^ Complex mixture of reduced products were formed in the reaction, but no target diol was observed in the reaction mixture by ESI-MS. ^e^ No conversion was observed.

## Experimental

### General

All 1,5-dicarbonyl compounds were synthesized following literature procedures [24,25]. InCl_3_·4H_2_O was dehydrated by reflux in SOCl_2_ prior to use, and all other chemicals used in the experiments were obtained from commercial sources and used without further purification. The reactions were carried out under an open atmosphere. Melting points were determined using an XT-4 apparatus and were not corrected. ^1^H- and ^13^C-NMR spectra were recorded in CDCl_3_ on a Bruker AVANCE DMX-500 spectrometer at 500 and 125 MHz, respectively. Chemical shifts are reported in ppm (*δ*), relative to an internal tetramethylsilane (TMS) standard. Mass spectra were recorded on a Bruker Esquire 3000plus mass spectrometer (Bruker-Franzen Analytik GmbH Breman, Germany) equipped with an ESI interface and ion trap analyzer. 

### General procedure of the intramolecular pinacol coupling reaction

1,5-Dicarbonyl compound (0.5 mmol), InCl_3_ (0.5 mmol), Al powder (1.8 mmol) and NH_4_Cl (0.5 g) were placed in a vessel, mixed solvent (H_2_O: EtOH=1:1, 4 mL) was added and the mixture was stirred at 70 ^o^C for 8 h. The completion of the reaction was monitored with TLC. After cooling down to rt, the mixture was extracted with ethyl ether (3×10 mL). The combined organic layer was dried overnight with anhydrous sodium sulphate. After removal of solvent, the residue was subjected to silica gel chromatography to give the corresponding product.

*1,2,4-Triphenylcyclopentane-1,2-diol* (**2a**). White solid; m.p. 133-135 ^o^C; ^1^H-NMR: *δ* = 7.34 (d, 4H, *J* = 5.5 Hz), 7.25 (t, 1H, *J* = 6.0 Hz), 7.00 (t, 10H, *J* = 8.0), 4.06-3.98 (m, 1H), 3.41 (s, 2H), 2.75 (dd, 2H, *J_1_* = 10.4 Hz, *J_2_* = 3.4 Hz), 2.52 (dd, 2H, *J_1_* = 8.9 Hz, *J_2_* = 5.0 Hz); ^13^C-NMR: *δ* = 144.4, 143.4, 128.8, 127.6, 127.5, 127.4, 127.3, 127.1, 85.8, 46.5, 38.7; IR (KBr, cm^-1^): 3420, 3310, 3026, 2924, 1496, 1278, 1191, 940. ESI-MS [M+Na]^+^: *m/z* 353. HRMS: Calcd. for C_23_H_22_O_2_Na [M+Na]^+^: 353.1512; Found, 353.1513.

*4-Phenyl-1,2-dip-tolylcyclopentane-1,2-diol* (**2b**). White solid; m.p. 155-157 ^o^C; ^1^H-NMR: *δ* = 7.32 (d, 4H, *J* = 5.7 Hz) 7.20 (t, 1H, *J* = 5.9 Hz), 6.87 (d, 4H, *J* = 8.0 Hz), 6.77 (d, 4H, *J* = 7.9 Hz), 4.02-3.97 (m, 1H), 3.28 (s, 2H), 2.72 (dd, 2H, *J_1_* = 10.2 Hz, *J_2_* = 3.5 Hz), 2.51 (dd, 2H, *J_1_* = 9.0 Hz, *J_2_* = 4.9 Hz), 2.09 (s, 6H); ^13^C-NMR: *δ* = 144.6, 140.5, 136.5, 128.8, 128.3, 127.3, 126.5, 126.4, 85.7, 45.8, 38.5, 21.2; IR (KBr, cm^-1^): 3467, 3301, 1922, 1514, 1281, 1190, 941; ESI-MS [M+Na]^+^: *m/z* 381; HRMS: Calcd. for C_25_H_26_O_2_Na [M+Na]^+^: 381.1825; Found, 381.1828.

*1,2-bis(4-Methoxyphenyl)-4-phenylcyclopentane-1,2-diol* (**2c**). White solid; m.p. 128-129 ^o^C; ^1^H-NMR: *δ* = 7.28 (d, 4H, *J* = 6.5 Hz), 6.83 (t, 2H, *J* = 8.0 Hz), 6.52-6.47 (m, 7H); 3.93-3.80 (m, 1H), 3.69 (s, 2H), 3.38 (s, 6H), 2.64 (dd, 2H, *J_1_* = 10.0 Hz, *J_2_* = 3.8 Hz), 2.43 (dd, 2H, *J_1_* = 9.0 Hz, *J_2_* = 4.9 Hz); ^13^C-NMR: *δ* =144.9, 128.9, 127.9, 127.2, 126.2, 119.0, 113.1, 112.8, 111.5, 85.6, 55.2, 45.2, 38.3. IR (KBr, cm^-1^): 3466, 3303, 2926, 1601, 1489, 1290, 1169, 1047, 962; ESI-MS [M+Na]^+^: *m/z* 413; HRMS: Calcd. for C_25_H_26_O_4_Na [M+Na]^+^: 413.1723; Found, 413.1714.

*1,2-bis(4-Chlorophenyl)-4-phenylcyclopentane-1,2-diol* (**2d**). White solid; m.p. 147-150 ^o^C; ^1^H-NMR: *δ*= 7.43-7.38 (m, 4H), 7.32 (t, 1H, *J* = 6.8 Hz), 7.05 (d, 4H, *J* = 8.6 Hz), 7.00 (d, 4H, *J* = 8.6 Hz), 4.14-4.06 (m, 1H), 3.38 (s, 2H), 2.76 (dd, 2H, *J_1_* = 10.0 Hz, *J_2_* = 3.9 Hz), 2.60 (dd, 2H, *J_1_* = 9.0 Hz, *J_2_* = 5.0 Hz); ^13^C-NMR: *δ* = 143.9, 141.7, 133.3, 129.0, 127.9, 127.8, 127.2, 126.7, 85.4, 45.5, 38.4; IR (KBr, cm^-1^): 3462, 3292, 1949, 1600, 1493, 1278, 1095, 939; ESI-MS [M+Na]^+^: *m/z* 421; HRMS: Calcd. for C_23_H_20_Cl_2_O2Na [M+Na]^+^: 421.0733; Found, 421.0717.

*1,2-Diphenyl-4-p-tolylcyclopentane-1,2-diol* (**2e**). White solid; m.p. 145-147 ^o^C; ^1^H-NMR: *δ* = 7.20 (d, 2H, *J* = 8.0 Hz), 7.11 (d, 2H, *J* = 10.0 Hz), 6.96-6.90 (m, 10H), 4.00-3.93 (m, 1H), 3.27 (s, 2H), 2. 71 (dd, 2H, *J_1_* = 10.2 Hz, *J_2_* = 4.0 Hz), 2.48 (dd, 2H, *J_1_* = 8.8 Hz, *J_2_* = 5.5 Hz), 2.27 (s, 3H); ^13^C-NMR: *δ* = 143.7, 141.6, 136.2, 129.8, 127.8, 127.4, 127.3, 126.7, 86.2, 46.1, 38.6, 21.5; IR (KBr, cm^-1^): 3470, 3300, 2923, 1516, 1369, 1058, 943; ESI-MS [M+Na]^+^: *m/z* 367. HRMS Calcd. for C_24_H_24_O_2_Na [M+Na]^+^: 367.1669; Found, 367.1661.

*4-(4-Chlorophenyl)-1,2-diphenylcyclopentane-1,2-diol* (**2f**). White solid; m.p. 142-145 ^o^C; ^1^H-NMR : *δ* = 7.29 (d, 2H, *J* = 8.1 Hz), 7.24 (d, 2H, *J* = 8.1 Hz), 6.94 (s, 10H), 3.97-3.93 (m, 1H), 3.46 (s, 2H), 2.66 (dd, 2H, *J_1_* = 10.7 Hz, *J_2_* = 1.6 Hz), 2.48 (dd, 2H, *J_1_* = 9.1 Hz, *J_2_* = 4.4 Hz); ^13^C-NMR: *δ* = 143.2, 142.9, 132.1, 128.9, 128.6, 127.6, 127.2, 126.4, 85.8, 45.4, 38.1; IR (KBr, cm^-1^): 3467, 3294, 2922, 1493, 1092, 1014, 945; ESI-MS [M+Na]^+^: *m/z* 387; HRMS: Calcd. for C_23_H_21_ClO_2_Na [M+Na]^+^: 387.1122; Found, 387.1122.

*4-(4-Methoxyphenyl)-1,2-diphenylcyclopentane-1,2-diol* (**2g**). White solid; m.p. 122-125 ^o^C; ^1^H-NMR: *δ* = 7.36 (d, 2H, *J* = 8.5 Hz), 7.25 (d, 2H, *J* = 9.5 Hz), 7.09-7.02 (m, 8H), 6.97 (d, 2H, *J* = 8.6 Hz), 4.10-4.03 (m, 1H), 3.86 (s, 3H), 3.39 (s, 2H), 2.80 (dd, 2H, *J_1_* = 10.2 Hz, *J_2_* = 3.9 Hz), 2.60 (dd, 2H, *J_1_* =8.8 Hz, *J_2_* = 5.4 Hz); ^13^C-NMR: *δ* = 143.2, 142.3, 131.9, 128.2, 127.6, 127.1, 126.5, 114.2, 86.1, 55.6, 46.0, 38.0; IR (KBr, cm^-1^): 3464, 3300, 2952, 1610, 1514, 1180, 1059, 938; ESI-MS [M+Na]^+^: *m/z* 383; HRMS: Calcd. for C_24_H_24_O_3_Na [M+Na]^+^: 383.1618; Found, 383.1608.

*4-(2-Chlorophenyl)-1,2-diphenylcyclopentane-1,2-diol* (**2h**). White solid; m.p. 132-135 ^o^C; ^1^H-NMR: *δ* = 7.48 (d, 1H, *J* = 8.0 Hz), 7.40-7.38 (m, 1H), 7.30-7.27 (m, 1H), 7.10-7.08 (m, 1H), 7.01-6.98 (m, 10H), 4.36-4.27 (m, 1H), 3.34 (s, 2H), 2.70 (dd, 2H, *J_1_* = 10.2 Hz, *J_2_* = 3.8 Hz), 2.56 (dd, 2H, *J_1_* = 8.7 Hz, *J_2_* = 5.4 Hz); ^13^C-NMR: *δ* = 143.2, 130.3, 129.4, 128.6, 127.8, 127.5, 127.4, 127.1, 126.7, 126.5, 85.6, 44.1, 36.7; IR (KBr, cm^-1^): 3549, 3462, 3061, 2981, 1601, 1489, 1083, 941; ESI-MS [M+Na]^+^: *m/z* 387; HRMS: Calcd. for C_23_H_21_ClO_2_Na [M+Na]^+^: 387.1122; Found, 387.1113.

*1,2,4-tri-p-Tolylcyclopentane-1,2-diol* (**2i**). White solid; m.p. 146-149 ^o^C; ^1^H-NMR: *δ* = 7.21 (d, 2H, *J* = 7.8 Hz), 7.12 (d, 2H, *J* = 7.8 Hz), 6.86 (d, 4H, *J* = 8.2 Hz), 6.76 (d, 4H, *J* =8.0 Hz), 3.95-3.91 (m, 1H), 3.31 (s, 2H), 2.66 (dd, 2H, *J_1_* = 10.1 Hz, *J_2_* = 3.7 Hz), 2.45 (dd, 2H, *J_1_* = 9.0 Hz, *J_2_* = 4.9 Hz), 2.30 (s, 3H), 2.12 (s, 6H); ^13^C-NMR: *δ* = 141.5, 140.6, 136.5, 135.8, 129.4, 128.2, 127.2, 126.5, 85.7, 45.8, 38.1, 21.3, 21.2; IR (KBr, cm^-1^): 3469, 3283, 3026, 2920, 1514, 1190, 1022, 943; ESI-MS [M+Na]^+^: *m/z* 395. HRMS: Calcd. for C_26_H_28_O_2_Na [M+Na]^+^: 395.1982; Found, 395.1974.

*4-(4-Fluorophenyl)-1,2-diphenylcyclopentane-1,2-diol* (**2j**). White solid; m.p. 163-165 ^o^C; ^1^H-NMR: *δ* = 7.68 (d, 2H, *J* = 8.0 Hz), 7.54 (d, 2H, *J* = 8.0 Hz), 7.05 (d, 10H, *J* = 5.1 Hz), 4.18-4.14 (m, 1H), 3.48 (s, 2H), 2.83 (dd, 2H, *J_1_* = 10.3 Hz, *J_2_* = 3.5 Hz), 2.65 (dd, 2H, *J_1_* = 8.9 Hz, *J_2_* = 5.0 Hz); ^13^C-NMR: *δ* = 147.3, 143.1, 127.7, 127.6, 127.3, 126.4, 125.8, 125.7, 85.7, 45.4, 38.6; IR (KBr, cm^-1^): 3450, 3185, 2930, 1510, 1089, 943; ESI-MS [M+Na]^+^: *m/z* 371; HRMS: Calcd. for C_23_H_21_FO2Na [M+Na]^+^: 371.1418; Found, 371.1423.
